# Inductive Heating Using a High-Magnetic-Field Pulse to Initiate Chemical Reactions to Generate Composite Materials

**DOI:** 10.3390/polym11030535

**Published:** 2019-03-21

**Authors:** Cordelia Zimmerer, Catalina Salazar Mejia, Toni Utech, Kerstin Arnhold, Andreas Janke, Joachim Wosnitza

**Affiliations:** 1 Leibniz Institute of Polymer Research Dresden e.V., Polymer Materials, Reactive Processing, 01069 Dresden, Germany; utech@ipfdd.de (T.U.); arnholdk@ipfdd.de (K.A.); andy@ipfdd.de (A.J.); 2 Hochfeld-Magnetlabor Dresden (HLD-EMFL), Helmholtz-Zentrum Dresden-Rossendorf, 01328 Dresden, Germany; c.salazar-mejia@hzdr.de (C.S.M.); j.wosnitza@hzdr.de (J.W.); 3 Institute of Solid State and Materials Physics, Electronically Correlated Matter, Dresden University of Technology, 01062 Dresden, Germany

**Keywords:** induction heating, high-magnetic-field, polycarbonate, bonding polymers, susceptor material

## Abstract

Induction heating is efficient, precise, cost-effective, and clean. The heating process is coupled to an electrically conducting material, usually a metal. As most polymers are dielectric and non-conducting, induction heating is not applicable. In order to transfer energy from an electromagnetic field into polymer induction structures, conducting materials or materials that absorb the radiation are required. This report gives a brief overview of induction heating processes used in polymer technology. In contrast to metals, most polymer materials are not affected by electromagnetic fields. However, an unwanted temperature rise of the polymer can occur when a radio frequency field is applied. The now available high-field magnetic sources provide a new platform for induction heating at very low frequencies, avoiding unwanted thermal effects within the material. Using polycarbonate and octadecylamine as an example, it is demonstrated that induction heating performed by a magnetic-field pulse with a maximum flux density of 59 T can be used to initiate chemical reactions. A 50 nm thick Ag loop, with a mean diameter of 7 mm, placed in the polymer-polymer interface acts as susceptor and a resistive heating element. The formation of urethane as a linker compound was examined by infrared spectroscopic imaging and differential scanning calorimetry.

## 1. Introduction

### 1.1. Induction Heating for Materials Processing

**Figure 1:**
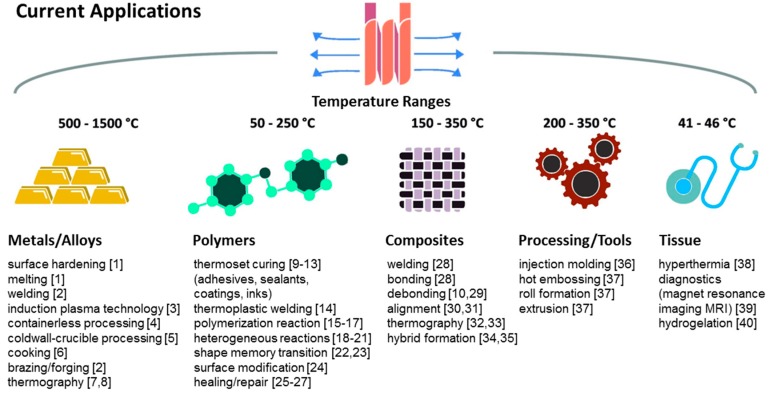
Different applications of induction heating for various materials and their processing temperatures.

Induction heating (IH) is used in industry, medicine, household, et cetera [1,2,3,4,5,6,7,8,9,10,11,12,13,14,15,16,17,18,19,20,21,22,23,24,25,26,27,28,29,30,31,32,33,34,35,36,37,38,39,40]. As non-ionizing radiation it is applied to trigger or to control physical processes and chemical reactions (see Figure 1). IH provides a high efficiency because electrically conductive materials are internally heated by induced currents.

In applying IH, no direct contact, e.g., to heating plates, is required [11]. IH can be considered as an alternative technology to thermal or radiation processing of plastics and polymers [16]. Further advantages, in polymer chemistry in particular, are environmental sustainability, the reduction or complete exclusion of solvents needed for the curing of coatings [16,28], and axing the use of a catalyst. This is caused by reaching a high temperature very quickly, a high productivity, and a reduction of cycle times [24]. IH provides great process variability, it allows heating of opaque composites [16], for joining complex components [34], and the joints are ready-to-use after processing. In comparison to conventional heating methods applied in polymer synthesis, IH leads to similar results for conversion rates, polymerization degree, and glass transition temperature of the formed polymer and the final adhesion of joints [20,24].

The rate of heating depends upon the frequency of the induced current. According to Faraday’s law of induction, the induced current is correlated to the frequency. Consequently, high-frequency electromagnetic fields, such as microwaves, are ideal for induction processes. On the one hand, in [41] a microwave-based rapid polymer processing technique is demonstrated to initiate chemical reactions and accelerate reaction kinetics, to change the diffusional behavior, and to generate gradients in polymer layers. On the other hand, the selective degradation of polymers was successfully applied to generate ordered crystalline frameworks by microwave heating [42].

Other IH approaches to plastics and composite materials are based on thermoset curing and thermoplastic bonding or welding. Other types of on-command reactions comprise polymerizations and reactions in heterogenic systems. Thus, the spatiotemporal control of IH makes it an interesting technology for traditionally engineered materials, especially for temperature-sensitive substrates [9,21,28], e.g., paper, wood, human tissue, hydrogels, plastics, and natural fiber reinforced composites. The ability for internally applied and localized heat, with high gradients, allows for processing at room temperature and also for thick samples by avoiding large thermal stresses, degradation, or deformation resulting in adhesive failures. Another benefit of IH is based on the possibility to detach and reassemble hybrid joints at the end of their product lifetime [29,34]. 

The aim of this study is to demonstrate the potential of electromagnetic field pulses with very high magnetic flux densities to join different polymers without induced thermal effects in the polymer bulk phases. As most applications of electromagnetic fields in polymer technology are based on high-frequency fields, general physical relations and selected examples of established approaches are first highlighted in the following two sub sections.

### 1.2. Interaction of Polymers with Electromagnetic Radiation

Common polymers are dielectric materials as they do not have any free charges. All electrons are bound or associated with atoms. However, when an electromagnetic field is applied, a slight displacement of the bound electrons is induced. The displacement creates small electric dipoles within the dielectric material. This process is called polarization where negative charges are displaced to the opposite direction of positive charges. Peter Debye developed a model describing the interaction of an electromagnetic field with dielectric media. According to the Debye model, polar entities of the dipoles tend to align parallel to the direction of the applied electromagnetic field. At lower frequencies, the dipoles have ample time to follow the vector of the electromagnetic field, generating a new electromagnetic wave. When the frequency increases, the dipoles become more and more unable to fully restore their original orientation. Thus, energy is absorbed by the dielectric material, as long as the electromagnetic field enforced motions of the polar entities take place. As charges are displaced from their equilibrium position within the molecule, polarization is related to the transfer of energy. How the dielectric material is polarized can be measured by the electric susceptibility (*Χ_e_*). The ability of the material to polarize reduces the electric field inside the material. The permittivity (*ε*) refers to the ability of the material to permit an electromagnetic field. The complex permittivity, also called dielectric constant, characterizes the interaction of an electromagnetic field with the material and is expressed as follows:

(1) $$\varepsilon =\varepsilon^{\prime }-i\varepsilon^{\prime \prime }.$$

While the real part of the dielectric constant (*ε*′) describes the propagation of the field, the imaginary part (*ε*″) relates to the energy absorbed by the material. The imaginary part is also often called “loss factor”. Polar polymers contain a relatively high number of permanent dipoles leading to a high loss factor. Non-polar polymers have a low loss factor. However, it should be noted that these polarization effects are relevant at frequencies between 0 Hz (steady field) and approximately 10^12^ Hz. At higher frequencies the permanent dipoles can no longer follow the frequency of the electromagnetic field and only electron distortion polarization is possible, referred to as infrared absorption. For most polymer materials, *ε*′ decreases monotonically from a high value at 0 Hz to lower values towards 10^12^ Hz. The loss factor (*ε*″) is zero at 0 Hz and at far infrared frequencies (>10^12^ Hz), showing a maximum at an intermediate frequency governed by the relaxation time. The behavior is the reason for heating when a high frequency (*f* = 10^6^–10^9^ Hz) electromagnetic field interacts with a polymer material. 

Materials that are used to absorb electromagnetic energy are often denoted as susceptors. Usually, susceptors convert the electromagnetic energy in thermal energy and are often used as a heating component for microwaves. Susceptors are an ideal transducer if a small volume within a polymer material has to be heated, e.g., to trigger a reaction or to modify specific material properties. 

**Figure 2:**
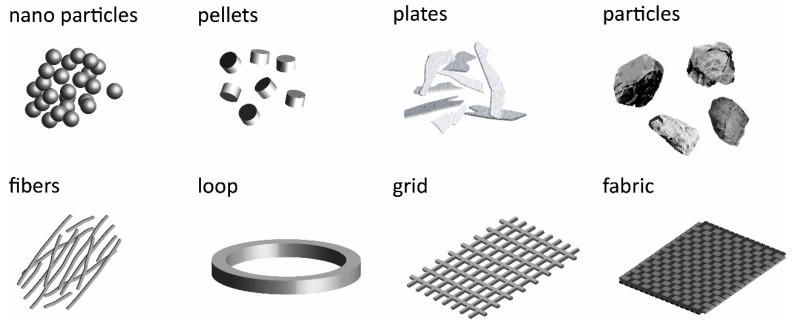
Different designs and materials used as susceptors (not to scale).

A selection of the most common susceptors, used in the different fields of polymer synthesis and processing, are represented in Figure 2. For high-frequency applications, metallic additives, metal meshes, ferro- or ferrimagnetic (nano) particles, and semiconducting carbon fiber fabrics [9] are applied. In all susceptors an external electromagnetic field induces a current, resulting in resistance heating. The frequencies are in the kHz and MHz range in order to induce energy. Recently, a low frequency high-magnetic-field (HF) pulse (59 T) was also successfully applied to induce a current in a thin metallic loop and to trigger a chemical reaction between Bisphenol-A based polycarbonate (PC) and a polyvinylamine [21,35]. 

For particulate susceptors forming a percolating network, the contact resistance at the particle or fiber junctions can be the dominating heating effect [9]. Important properties of the susceptor are the (a) electrical and thermal conductivity, (b) heat capacity, (c) form and dimension, and (d) surface morphology. Graphene might be a well-suited candidate as particulate susceptor, especially for structures with a very low thickness and the need for transparency [43,44]. However, currently available graphene structures are not conductive enough to induce sufficient energy [45]. The overall heating properties of particulate embedded susceptors are difficult to predict because these various factors, (a) to (d), are related to each other by complex behavior [2]. A poor adhesion with the surrounding polymer can create defects within the polymer and lead to residual stress at the interfaces and, probably, excessive deformation [32]. 

Table 1 summarizes susceptor materials, energy conversion in electromagnetic fields, and thermal processes. The listed heating effects depend on the applied frequency of the electromagnetic field. Some effects, e.g., the ring effect, depend on the experimental set-up, the distance between sample and the type of coil. On the other hand, the skin effect has not been considered for low frequencies. For HF IH applications, loops are applied as susceptors and placed into the homogeneous field of the coil bore volume. This sample-coil set-up results in negligible proximity and ring effect. 

### 1.3. Chemical Reactions at Polymer-Polymer Interfaces

The term reaction promoter is used if IH is utilized to initiate chemical reactions surrounding the susceptor [2]. To the best of our knowledge, polymerization reactions for curing processes are the most common fields to initiate a chemical reaction by IH. The polymerization reaction can be performed to cure high-temperature thermosets [11,16] as well as for polymerization reactions at relatively low temperatures [15,16,17,26,27]. A further application was published about the decomposition of an organic compound to release nitrogen gas [18]. A reactive joining to form a polymer hybrid component [48] is based on the triggering of a chemical reaction by IH in HF. The reactive joining and hybrid formation can be divided into the following five process steps: (i) Susceptor placement, (ii) electromagnetic induction, (iii) heating, (iv) chemical reaction, and (v) cooling. A contact between the polymers is required [34]. An interpenetration of polymer chains can support the formation of a compact interphase. Another aspect concerning the triggering of chemical reactions is realized in the debonding processes or a reassembling to detach joined materials and composites by a thermal degradation reaction [29,49].

**Figure 3:**
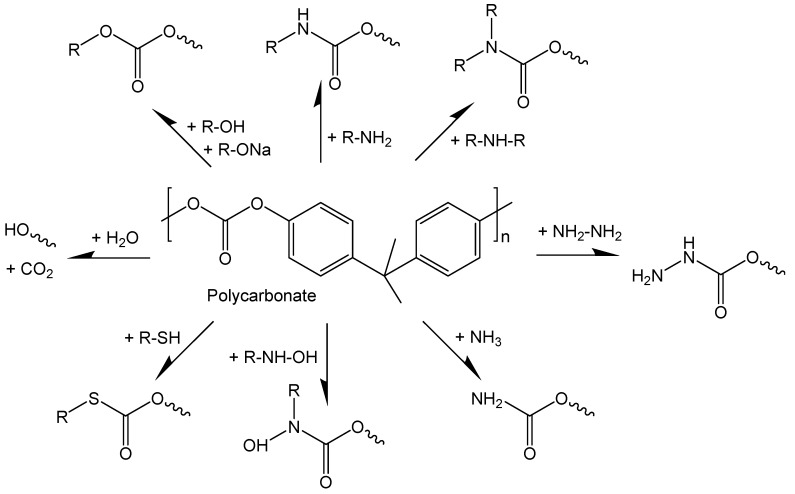
Scheme of chemical reactions for PC with different components.

A common thermoplastic, PC, has found wide-spread applications as a cheap, transparent, and nonpolar plastic material. Its molecular structure makes it a well-suited candidate for reactive joining. The carbonate groups can act as a nucleophilic reaction center for the attack by different functional and electrophilic groups. Selected compound classes, which are reactive to PC, and newly formed coupling groups of products are shown in Figure 3.

A former published experiment [21,48] described the chemical reaction of polycarbonate (PC) with a polyelectrolyte polyvinylamine (PVAm) and proved for the first time the new bond formation. The reactivity is based on the presence and density of functional groups and their steric proximity. The requirement to realize an appropriate distance and orientation is the mobility of reactive groups or the entire molecule. In the solid state, mobility is reduced by intermolecular interactions and is smaller by orders of magnitude than in the liquid or dissolved state. For solids, the thermodynamically preferred crystalline state is less reactive through stronger short and long-range interactions. The amorphous state is marked by larger distances between chains and weaker interaction. Both PC and PVAm are both amorphous polymers and PVAm has the highest density of the primary amino groups.

The understanding of the reaction mechanism of PC with primary amines was experimentally investigated by IR spectroscopy. In general, the 2-reactive-component system leads to a copolymerization reaction. Urethane groups as linker structures are formed, leading to a substance to substance bond which results in a stable and permanent hybrid material. In order to gain a deeper insight into the solid-state chemical reaction mechanism taking place in the interfacial layer, a further system consisting of PC with the crystalline octadecylamine (ODA) was investigated [50]. As a low molecular weight compound, ODA possesses different steric demands, higher movability, but clearly reduced amino group density.

## 2. Materials and Methods

### 2.1. Chemicals

PC Makrolon^®^ 2245 was received from Bayer AG (Leverkusen, Germany). Octadecylamine (ODA) with 97% purity and silver were supplied by Sigma Aldrich (Munich, Germany). Chloroform (99% purity) and toluene were received from Acros Organics (Geel, Belgium). Ethanol absolute was received from VWR International (Dramstadt, Germany). 

### 2.2. Sample Preparation

Polymer samples were prepared on an IR transparent calcium fluoride (CaF2) window with a thickness of 1 mm and 10 mm in diameter (Korth Kristalle GmbH, Altenholz, Germany). The pure CaF2 window was rinsed with chloroform, absolute-ethanol, and dried afterwards with air. Subsequently, a PC solution of 10 g/L, solved in a 1:1 mixture of chloroform and toluene, was spin coated on the pure surface of the substrate. The resulting PC film thickness amounts to approximately 46 µm. Afterwards, a 50 nm thick silver ring with an inner diameter of 6 mm and an outer diameter of 8 mm was vapor deposited onto the PC layer, induced in a high vacuum chamber (*p* = 1 × 10^−6^ mbar) Creamet 300V2 from Creavac GmbH (Dresden, Germany), at a constant rate of 1 nm/s until the quartz crystal microbalance registered the desired thickness. The Ag ring acts as susceptor. The sample had an 18 cm distance to the Ag source and was kept at room temperature. ODA was solved in ethanol-absolute. The concentration of the ODA solution was adjusted to 30 mmol/L. The solution was used to spin-coat an ODA layer onto the PC-layer/silver ring at 500 rpm with a ramp of 1000 rpm/s for 30 s time at final speed.

### 2.3. High-Magnetic-Field Induced Heating Experiment

The pulsed magnets are energized using a 50 MJ/24 kV modular capacitor bank [51,52]. The magnets are installed in individual pulse cells and electrically separated from each other. Liquid nitrogen is used to cool the magnets down to 77 K to reduce the ohmic resistance. Different pulsed-field magnets are available at the Dresden High Magnetic Field Laboratory (HLD). The magnet used is made out of soft copper wire with Zylon^®^ fiber reinforcement. Information on magnet details can be found elsewhere [53].

**Figure 4:**
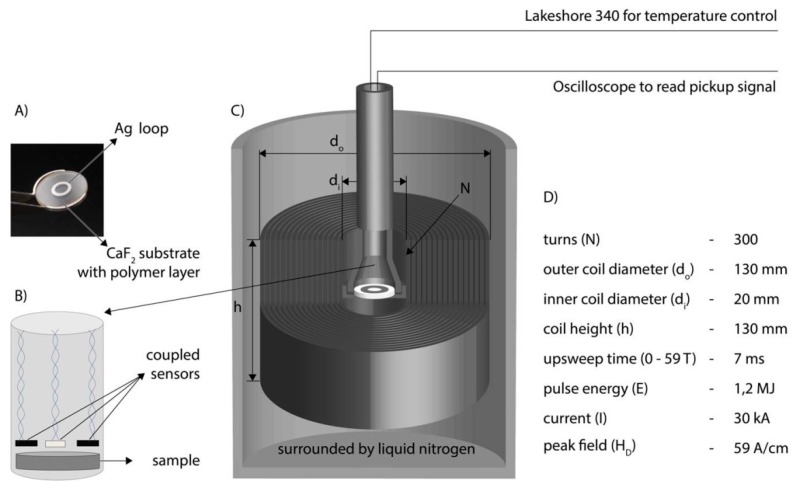
Schematic drawing of experimental set-up and parameters for magnetic-field pulse. (**A**) Photograph of a sample with silver ring on a spectroscopic substrate. (**B**) Schematic drawing of the thermo- and magneto-sensors near the sample surface. (**C**) Position of the sample and the pulse setup. (**D**) Specifications and coil parameters.

In this study, a so-called KS21 magnet with a 20 mm bore was used (coil parameters are listed in Figure 4D). In this specific magnet, a voltage of 22 kV corresponds to a maximum magnetic field of *B_max_* = 59 T with a total amount of energy of 1.2 MJ. The pulse duration is approximately 25 ms. 

Figure 4 shows a schematic drawing of the experimental set-up. A home-built sample holder made out of Polyetheretherketone was used to place the sample (Photograph in Figure 4A) in the center of the magnetic field, Figure 4C. The sample was kept at room temperature during the pulse and temperature was controlled and recorded permanently (Figure 4B). The temperature was controlled using a Lakeshore 340 temperature controller.

The value of the magnetic field was determined by measuring the induced voltage in a calibrated pick-up coil. The induced voltage was recorded by a digital oscilloscope, Yokogawa DL750 (or DL850). Data was stored and later integrated numerically to determine the magnetic-field intensity as a function of time.

### 2.4. Atomic Force Microscopy

The measurements in the atomic force microscopy were taken in the peak force tapping mode by a Dimension FASTSCAN (Bruker-Nano Surfaces Division, Santa Barbara, CA, USA). A silicon nitride sensor, FASTSCAN-B (Bruker, USA), with a nominal spring constant of 1.8 N/m and tip radius of 5 nm was used. The set-point was 0.015 V. The offline analysis was performed by using NanoScope Analysis v1.90 (Bruker-Nano Surfaces Division) software.

### 2.5. Resistance Measurements

**Figure 5:**
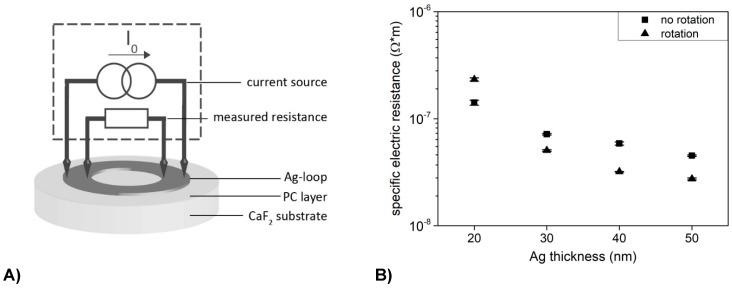
(**A**) Experimental set-up for determination of the resistance of the Ag loop. (**B**) Measured specific electric resistance depending on Ag loop thickness. Variation of evaporation process contained static and rotating samples during Ag deposition.

A digital multimeter DMM 2001, from Keithley Instruments (Germering, Germany), was used to measure the resistance of the susceptor loop. The resistance was measured by a constant current. Figure 5A illustrates the measurement set-up. In order to eliminate line and transition resistances from measurement objects with low resistance values, 4-wire measurement technology was used, i.e., constant current supply and voltage measurement are carried out via separate lines. The DMM 2001 covers a total measuring range between 1 µΩ and 1 GΩ. For the low-impedance loops, only the lowest measuring range 1 µΩ to 20 Ω is of interest, in which the measurement objects are fed with a constant current of 9.2 mA. This set-up allows two half rings to be measured in parallel. Assuming a homogeneous structure, the ring resistance *R_Ring_* is calculated from the measured resistance *R_mess_* as follows: Under the assumption that the contact-resistances are equal, the total resistance of the loop has the fourfold value of the measured resistance. For the thinnest Ag loops of 10 nm no resistance was measurable. 

### 2.6. Infrared Spectroscopic Imaging

FT-IR spectroscopic images were collected in transmission mode using a FT-IR spectrometer Vertex 70 coupled with an infrared microscope, Hyperion 3000 (both from Bruker Optics GmbH, Ettlingen, Germany), and a 64 × 64 MCT focal plane array detector. The 15-fold Cassegrainian objective, with a numerical aperture of 0.4, imaged a sample area of approximately 175 × 175 µm^2^. 

Spectra collection, data treatment, and evaluation are described in detail elsewhere [21].

### 2.7. Differential Scanning Calorimetry

Differential Scanning Calorimetry (DSC) was used to study the thermal behavior of PC and ODA. DSC curves were recorded by a Q2000 DSC device (TA-Instruments, New Castle, NJ, USA) in the temperature range between −80 °C and 200 °C, at a heating rate of 2 K/min modulated with ±0.31 K/40 s. The neat components PC and ODA were milled to powder. Each DSC measurement was carried out in Tzero aluminum crucibles with a sample mass of approximately 12 mg under nitrogen flow. Measurements of seven mixtures were performed with varying mass ratios of PC and ODA. For mixtures, the diligent milled samples were weighed directly into the DSC pans according to the desired mixing ratio. After closing the pans, they were shaken manually to obtain a thorough mixing.

## 3. Results and Discussions

### 3.1. Chemical Reaction between PC and ODA

**Figure 6:**
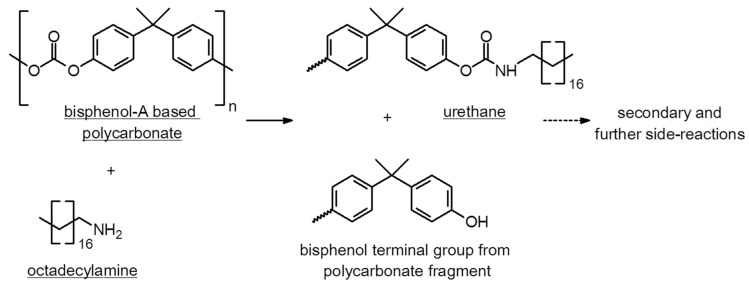
First step of chemical reaction between polycarbonate and octadecylamine, which shows the initial formation of urethane and the remaining chain fragment of polycarbonate. Secondary reactions are described elsewhere [48].

The interfacial reaction between PC and ODA indicates a controlled mechanism for the formation of urethane linkages (Figure 6) and directly influences the final joint strength. It is controlled by the temperature gradient of susceptors during IH and heat dissipation between the newly generated interphase and both bulk-hybrid components, PC and ODA. 

Since ODA is a non-polymeric amine, its reactivity is expected to be higher than for polymeric amines, such as polyvinylamine. The thermal-reactive behaviors of PC and ODA were investigated for different mixtures and compared with the neat PC and neat ODA. 

**Figure 7:**
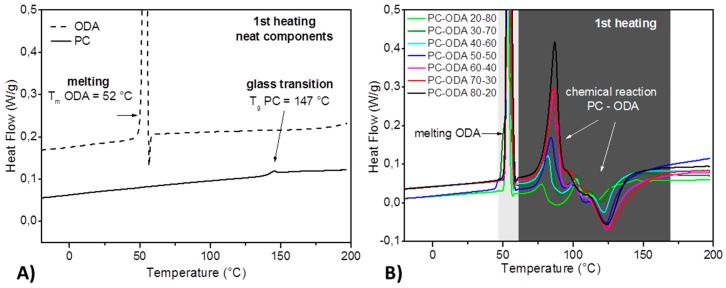
(**A**) DSC curves of neat ODA and neat PC. (**B**) DSC curves of seven mixtures from PC and ODA with mass ratios PC/ODA, as denoted in the legend.

For neat ODA, Figure 7A shows the heat flow depending on temperature. Within the temperature range, the melting process is clearly revealed. The glass transition of the neat PC was determined at 147 °C. If mixtures of PC and ODA are heated (Figure 7B), immediately after the melting of ODA, an endothermic reaction starts. The first-heating signal above 100 °C is assigned to secondary, exothermic chemical reactions. The measurement of the mixture with a mass ratio of PC/ODA 20/80 results in the only DSC curve (light green), showing four signals as follows: Melting of the ODA, the endothermic reaction between PC and ODA, the exothermic reaction, and the glass transition of PC. This points to the presence of the two educt phases and two different product phases. In all other mixtures, PC is completely converted or the remaining PC fragments cannot form a stable phase.

### 3.2. Estimation of the Induced Temperature Change

The induced voltage (*U*) in a ring of radius (*r*) according to Faraday’s law of induction is as follows:

(2) $$U\left(t\right)=-\left(\pi r^{2}\frac{dB}{dt}\right)\;,$$

where *dB*/*dt* is the temporal change of the magnetic field. The energy dissipated in the metal ring is as follows:

(3) $$E=\int \frac{U{\left(t\right)}^{2}}{R}dt,$$

where *R* is the resistance of the ring, which can be expressed as follows:

(4) $$R=\frac{2\pi r}{A}\rho ,$$

where *ρ* is the electrical resistivity. Assuming that the energy completely converts into thermal energy (*E*), the following relation can be made:

(5) E=cmΔT,

where *c* is the specific heat, m is the mass, and Δ*T* is the change in the temperature. Combining the equations, we get an induced temperature change as follows:

(6) $$\Delta T=\frac{r^{2}}{4\rho cD}\int {\left(\frac{dB\left(t\right)}{dt}\right)}^{2}dt,$$

with the density (*D*)

(7) D=m/(2πrA).

**Figure 8:**
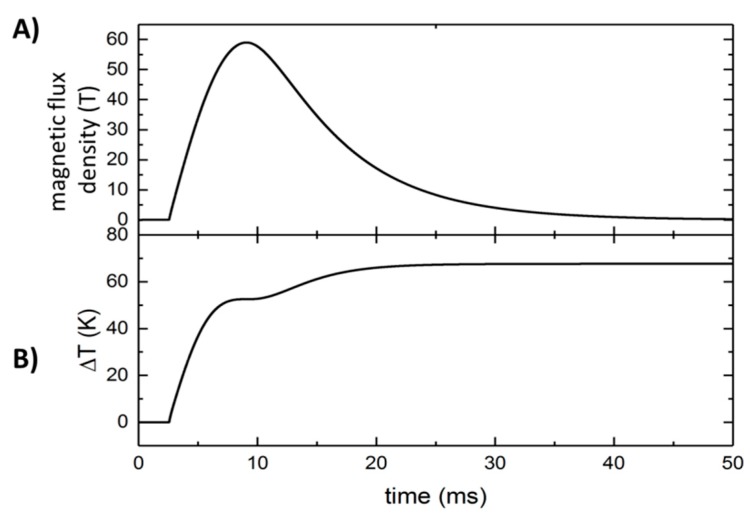
(**A**) Temporal magnetic-flux profile of the field pulse. (**B**) Heating of the Ag loop (50 nm thickness) with bulk metal properties caused by IH.

According to the values reported in Table 2, the induced temperature change on an Ag loop with an average radius of *r* = 3.5 mm (inner diameter 6 mm, outer diameter 8 mm) due to a 59 T/25 ms pulse, is estimated to be about 67.5 K, as shown in Figure 8B. 

Figure 8 shows the magnetic-flux density vs. the time for the field pulse applied in this experiment, together with the calculated temperature of the Ag ring due to electromagnetic induction.

The Ag susceptor loop has a thickness of 50 nm and a resistance of 15 Ohm. This is a very good compromise and also the lower limit of the thickness. A larger thickness would be more disadvantageous in respect to reactions across an ultrathin interface. A thickness of less than 50 nm leads to a higher resistance of the ring and the energy dissipation is too small. The final temperature of 67.5 °C is sufficient to trigger the reaction between PC and ODA.

### 3.3. Morphology of the Ag Loops

**Figure 9:**
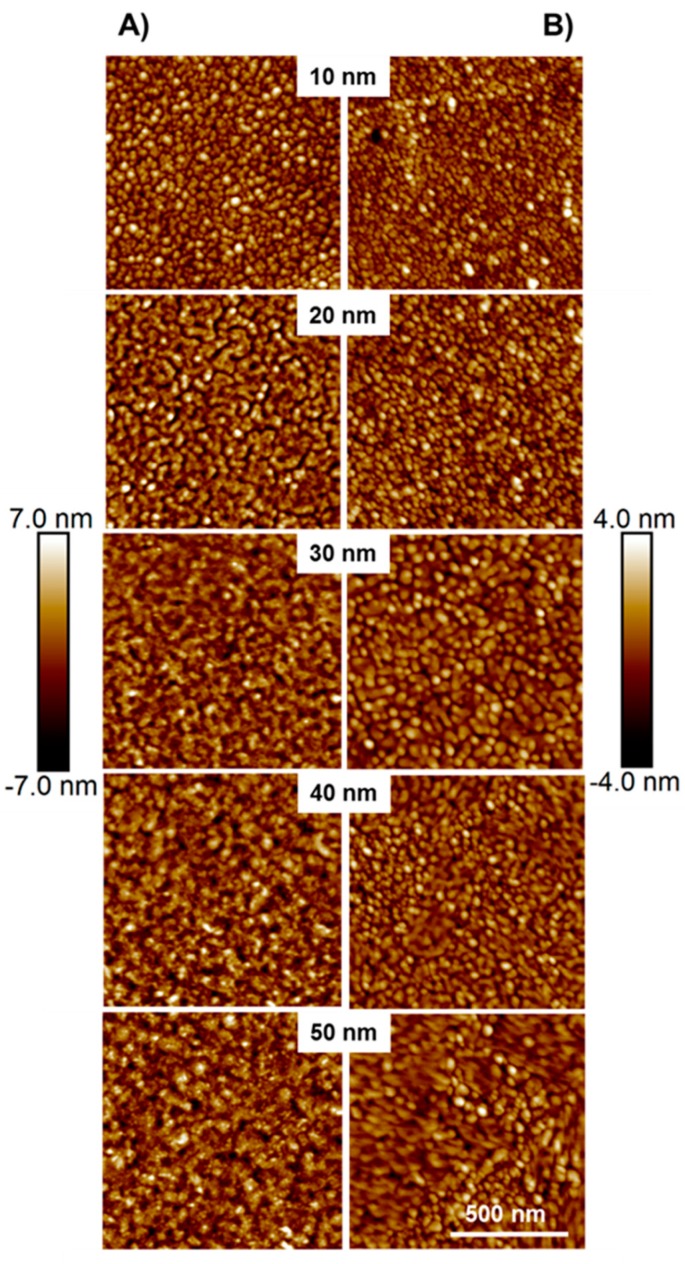
AFM topography images (1 µm × 1 µm) of evaporated Ag layers with different thickness on PC. Column (**A**) shows evaporation with no sample movement during deposition and column (**B**) shows evaporation with rotating the samples during deposition. The z-scales correspond to the images in the (**A**) or in the (**B**) column, respectively.

The Ag layer shows a granular structure which also controls the electrical resistance. The morphology is further influenced by the deposition process. Figure 9 shows the AFM images for different Ag ring thicknesses. The granular structures, shown in Figure 9 column A, were captured from samples without rotation during the evaporation process. Images in Figure 9 column B were recorded from samples with rotation during the evaporation process. The mean roughness *S_a_* (mean value from all 5 images) for the samples evaporated without rotation is 1.51 ± 0.01 nm and 0.81 ± 0.06 nm for the samples evaporated with rotation.

The AFM (z-scale) records a smoother surface for the evaporation with sample rotation. For 10 nm Ag layers, individual spherical clusters dominate the morphology. For thicker layers, morphology looks more like regular layers with sinkholes and trenches. Comparing both evaporation methods, there is no clear difference in the size or shape of the clusters for each individual thickness. The morphology of the Ag layers does not change significantly when the deposited Ag layer thickness increases. All images show a clear percolation of the Ag clusters and point to a conductive layer formation. For a smoother layer a better current distribution is expected. 

The resistance measurements (see Figure 5B) reveal a decrease in resistivity by increasing thickness of the Ag layers. The thicker the Ag loops the higher is their IH efficiency. The sample rotation further reduces the electrical resistance of the granular structures. 

However, the chemical reaction can only be initiated at the interface. As known from literature, the thickness of polymer interfaces is often in the range of a few nanometers. For thicker susceptor structures the bulk material of the components will be proportionally more heated. This heat is no longer available to trigger the interfacial reaction. 

### 3.4. Verification of Chemical Reaction by FTIR Spectroscopic Imaging

Reaction between PC and ODA due to electromagnetic induced heating of the Ag loops was spectroscopically evaluated as described for the polymeric system in [22]. 

**Figure 10:**
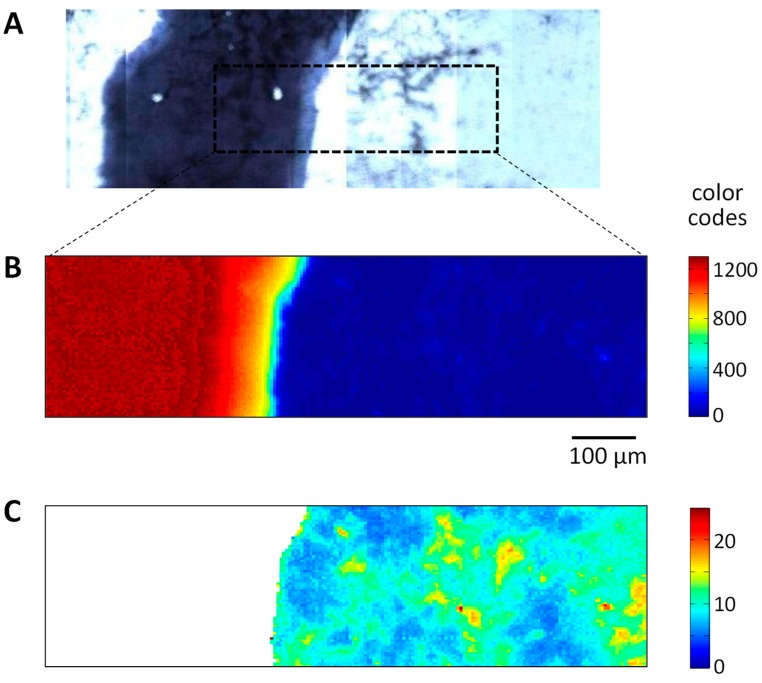
(**A**) Light microscopic image of the sample. The Ag ring appears as a dark area on the left side of the sample. The dotted rectangle indicates the sample area measured by FT-IR imaging. (**B**) FT-IR spectroscopic image. The contrast is calculated by integration of absorbance values between 950 and 2000 cm^−1^. (**C**) Selected FT-IR images of the inspected area. White pixels indicate spectra eliminated from the data set.

**Figure 11:**
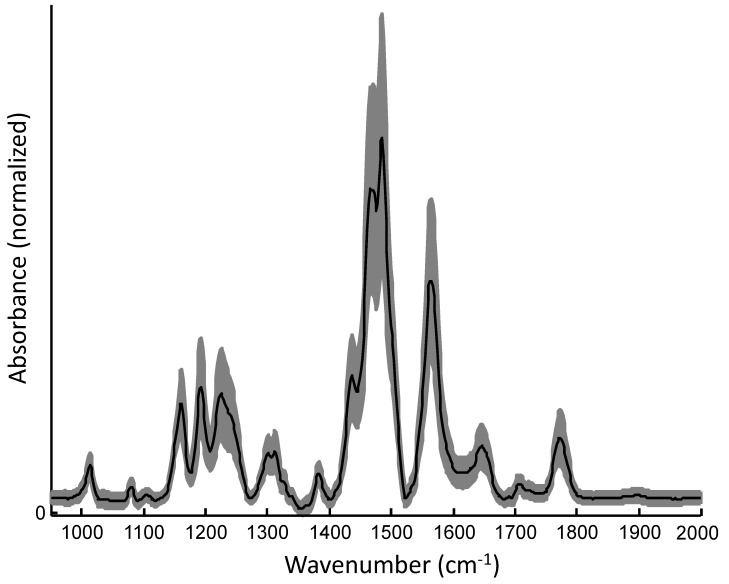
Mean spectrum (black) and standard deviation (gray) calculated from the data set of selected spectra.

Figure 10 shows the microscopic and IR spectroscopic image of the sample. The Ag ring appears in the microscopic image (Figure 10A) as a black area. Contrast of the IR spectroscopic image was calculated by the integration of absorbance values (Figure 10B). As the Ag ring reflects IR radiation it appears in red and brown colors, while the PC-ODA layer system is dominated by blue pixels. Spectra that correspond to the Ag ring were eliminated from the data set. The IR spectroscopic brightfield image IR (Figure 10C) reveals an inhomogeneous formation of the PC-ODA layer. The spectra of the pre-processed data set show a variation across the whole sample. This is also illustrated by the plot of the mean spectra and the standard deviation, represented in Figure 11. The spectral profile exhibits the typical bands of PC and ODA, assigned and discussed in the previous paper [48,50]. 

**Figure 12:**
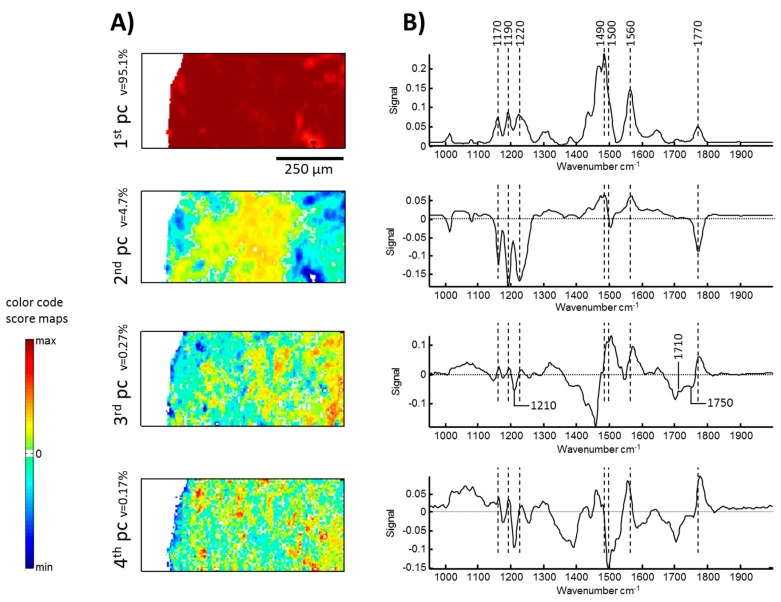
Principal component (pc) analysis of FT-IR Images recorded from a PC-ODA layer sample after exposure to a pulsed high-magnetic field. (**A**) Represents score maps and (**B**) shows the corresponding loading plots for each individual principle component.

The results of principal component analysis (PCA) are represented in Figure 12. As the PCA was performed on the raw matrix the loading plot of the 1st principal component (pc) represents the mean spectrum, showing a number of specific and characteristic signals of PC and ODA (see Supplementary Materials Tables S1–S4). The strongest signal between 1400 and 1500 cm^−1^ is a composition of CH_x_ deformation and ring vibration modes assigned to ODA and PC, respectively. ODA has another strong signal at 1560 cm^−1^, associated with vibrations of amino groups. The triplet between 1150 and 1250 cm^−1^ arises from C–O–C and O–C(O)–C vibrations of PC. Finally, the vibration mode of the carbonyl group of PC appears at 1770 cm^−1^. The score map shows predominantly red colors, indicating an overall homogeneous formation of the PC/ODA layer. The second pc points to variations within the PC and the ODA layer. Spectral features of PC appear as negative signals in the loading plot. While the pattern of negative signals in the loading plot of the 2nd pc corresponds very well with the spectrum of PC, positive signals indicate ODA. In the corresponding score map, yellow and orange pixels, as well as blue and green pixels, form larger areas and point to variations between the PC and ODA layers. The score map of the 3rd pc shows a small strip of dark green and blue pixels on the Ag ring. The corresponding loading plot exhibits a number of negative signals which are all associated with the expected reaction product of urethane. The most prominent signals are located at 1470 cm^−1^ and 1700 cm^−1^. The first relatively broad signal is a composition of NH, CN, and CH_x_ vibration modes. The signal around 1700 cm^−1^ is assigned to C=O vibration of the urethane group. In addition, positive signals represent PC and ODA. Negative values (blue and green pixels) in the score map indicate their conversion to the product. The formation of urethane is underlined by the 4th principal component. Besides the signals at 1700 cm^−1^ and 1220 cm^−1^, the relative strong signals centered at 1396 cm^−1^ (which is associated with CH_x_ groups), point again to the formation of urethane. Blue pixels in the infinity of the Ag ring reveal the conversion of PC and ODA. However, the overall variance is small and some other variations across the inspected area are present, making a full interpretation of the 4th principal component difficult. 

## 4. Conclusions

High-magnetic-field sources enable the development of novel techniques for polymer engineering. High energies can be transferred into a limited volume, such as an interface layer, within a very short period of time, while avoiding thermal effects in the polymer bulk phases. Strong electromagnetic fields are an outstanding platform for the development of new polymer composite and hybrid materials. In contrast to electromagnetic fields with radio frequency, the magnetic pulse does not cause thermal effects in the polymer materials. 

In this report it is demonstrated that a chemical reaction between PC and ODA can be initiated by electromagnetic induction when a 50 nm thin Ag loop is placed at the interface of both components. Just a single magnetic pulse of approximately 59 T leads to a local heating of the Ag susceptor, resulting in the formation of urethane groups from PC and ODA. The linker structures were probed by IR spectroscopic imaging. 

This study demonstrates the application potential of low-frequency but high-magnetic-field pulses in polymer technology and offers, in particular, new applications for the development of novel polymer composites and hybrids. The scalability of the experiments is coupled to the availability of high magnetic fields. Basically, the new techniques can be scaled up to samples several centimeters in size. 

## Figures and Tables

**Table 1:** Thermal processes in susceptor materials.

Heating Effect		Susceptor Material	Remarks	Literature
resistive heating	eddy current	electrically conductive		[2,6,21]
junction heating	local dielectric heating	particulate, electrically conductive	various factors in complex relationship	[2]
magnetic polarization	hysteresis heating	particulate, ferro- and ferrimagnetic	various factors in complex relationship	[3]
skin effect	current flow at outer surface	electrically conductive	increases with frequency	[5,46]
proximity effect	current crowding	electrically conductive	increases with frequency, thermal hot spot formation	[47]
edge effect	diffraction on arras	electrically conductive	defined by susceptors geometry	[9]
ring effect	variation in field line density	all susceptor materials	inhomogeneous field, which depends on the coupling distance	[2,47]

**Table 2:** Physical properties of silver (Ag) from [54].

Ring Material	Specific Heat (J/kg K)	Electrical Resistivity (Ω m)	Density (kg/m^3^)
Ag	235	1.6 × 10^−8^	10.5 × 10^3^

## Supplementary Materials

The following are available online at https://www.mdpi.com/2073-4360/11/3/535/s1, Table S1–S4: FTIR band assignments.
