# Supplementary material for: Inductive Heating Using a High-Magnetic-Field Pulse to Initiate Chemical Reactions to Generate Composite Materials
Source: Polymers (Basel). 2019 Mar 21;11(3):535. doi: 10.3390/polym11030535 (PMC6473677; doi:10.3390/polym11030535)
Supplement: Supplementary file 1 [file polymers-11-00535-s001.pdf]

IR Identification of PC, ODA, and Their Reaction Products

Abbreviations for band assignments

ν…stretching vibration
δ…deformation vibration
ω…wagging vibration
ρ…rocking vibration
γ…scissoring vibration
as…asymmtric
s…symmetric
o.o.p….out of plane
i.p….in plane

Abbreviations for band intensities

vw…very weak; w…weak; m…medium; strong; vs…very strong

Table S1. Assorted band assignments of Bisphenol-A based Polycarbonate (PC).

Wavenumber (cm^{-1})	Band assignment	Intensity	Molecular structure	References
3046	ν CH	w	aromatic ring (Ar)	[1,3]
2970	ν_{as} CH	m	CH_3	[1,3]
2870	ν_s CH		CH_3	[3]
1775 (dubl. 1785 trans-cis, 1767 trans-trans)	ν C=O	s	-O-(C=O)-O-, Ar-O-C(=O)-O-Ar	[2,4,6]
1600	δ_s CH, ν_{ip} CC ν CC	vw	aromatic ring	[3–5]
1505	ν C-C δ_{as} CH, Dublett, ν CC	s	aromatic ring	[2,3]
1462	δ CH, ν C=C, $\delta_{as,\ i.p.}$ CH	vw	CH_3	[3,5]
1409	$\delta_{as,\ i.p.}$ C-H	vw	CH_3	[3]
1388	δ_s CH, $\delta_{s,\ i.p.}$ CH	vw	CH_3	[5,7]
1365	δ_{as} CH, Dublett, $\delta_{s,\ i.p.}$ CH	vw	CH_3	[3,5]
1227	ν_{as} COC, δ C=O, ν O-C(O)-O	vs, broad	-O-(C=O)-O-, aromatic ring	[3,5,6]
1191	ν_{as} O-C(O)-O, $\omega_{i.p.}$ CH,	vs	-O-(C=O)-O-,	[3,5,6]
1168	ν O-(C=O)-O, ν COC, C-C, $\omega_{i.p.}$ CH	s	-O-(C=O)-O-, aromatic ring	[1,3,5,6]
1102	$\delta_{i.p.}$ CH	w	aromatic ring	[3]
1080	γ CCC, δ CH	m	aromatic ring	[2]

1009	ν_s OCO, ν OC	s	-O-(C=O)-O-, aromatic ring	[1–3,5]
884	ν C-C, $\delta_{o.o.p.}$ CH	w	C-CH_3	[3,5]
830	$\delta_{o.o.p.}$ CH, ω CH, $\gamma_{o.o.p.}$ CH	m	aromatic ring	[2,3]

1. Dechant, J. *Ultrarotspektroskopische Untersuchungen an Polymeren*; Akademie-Verlag: Berlin, Germany, 1972.
2. Kraus, R.G.; Emmons, E.D.; Thompson, J.S.; Covington, A.M. *J. Polym. Sci Part P Polym. Phys.* **2008**, *46*, 734–742.
3. Asensio, R.C.; Moya, M.S.A.; de la Roja, J.M.; Gomez, M. *Anal. Bioanal. Chem.* **2009**, *395*, 2081–2096.
4. Heymans, N.; Dequenne, B. *Polymer* **2001**, *42*, 5337–5342.
5. Lee, S.-N.; Stolarski, V.; Letton, A.; Laane, J. *J. Mol. Struc.* **2000**, 52119–52123.
6. Sweileh, B.A.; Al-Hiari, Y.M.; Kailani, M.H.; Mohammad, H.A. *Molecules* **2010**, *15*, 3661–3682.
7. Abbate, M.; Martuscelli, E.; Musto, P.; Ragosta, G.; Scarinzi, G. *J. Polym. Sci Part B Polym. Phys.* **1994**, *32*, 395–408.

Table S2. Assorted band assignments of Octadecylamine (ODA).

Wavenumber (cm^{-1})	Band assignment	Intensity	Molecular structure	References
3331	ν_{as} NH	s	NH_2	[1]
3255	ν_s NH	w	NH_2	[1]
3173	2δ NH overtone Amide II	m		[2]
2954	ν_{as} CH	m	CH_3	
2917	ν_{as} CH	vs	CH_2	[3]
2848	ν_s CH	s	CH_2	[4]
1647		w		
1608		m		
1565	Amide II	m		[2,4]
1485	δ_{as} CH	m	CH_3	[5]
1465	δ_{as} CH	m-s	CH_2	
1437	δ_s CH	w-m	CH_2	[3]
1380	δ_s NH	w	CH_2	[3]
1307		w		
1150		w		
1113		w		
1060	ν CN	m		
1045		w		
894	ρ CH	m	CH_2	[3]
719		w		

1. Sokoll, R.; Hobert, H. *J. Chem. Soc. Faraday Trans. I* **1986**, *82*, 1527–1535.
2. Spange, S.; Wolf, S.; Simon, F. *Progr. Colloid Polym. Sci.* **2006**, *132*, 110–116.
3. Rivas, B.L.; Seguel, G.V.; Geckeler, K.E. *Die angewandte Makromolekulare Chemie* **1997**, *251*, 97–106.
4. Das, P.K.; Ruzmaikina, I.; Belfiore, L.A. *J. Polym. Sci Part B: Polymer Phys.* **2000**, *38*, 1931–1938.
5. Kraus, R.G.; Emmons, E.D.; Thompson, J.S.; Covington, A.M. *J. Polym. Sci Part P Polym. Phys.* **2008**, *46*, 734–742.

Table S3. Assorted band assignments of Bisphenol-A (BPA).

Wavenumber (cm^{-1})	Band assignment	Intensity	Molecular structure	References
3350	ν_{as} OH	s, broad	associated, H-bond	[1]
3065	ν CH	w	aromatic ring	[2]
3029	ν CH	w	aromatic ring	[1]
2966	ν_{as} CH	s	CH_3	[1]
2871	ν_s CH	s	CH_3	[1]
1612		s-m	p-substituted aromat	[1]
1598		s-m	p-substituted aromat	[1]
1511		s		[1]
1455		s-m		
1363		m	C-CH_3	[1]
1178		s		[1]
826		m		[1]

1. Fei, B.; Chen, C.; Peng, S.; Zhao, X.; Wang, X.; Dong, L. *Polymer Int.* **2004**, *53*, 2092–2098.
2. Abbate, M.; Martuscelli, E.; Musto, P.; Ragosta, G.; Scarinzi, G. *J. Polym. Sci Part B Polym. Phys.* **1994**, *32*, 395–408.

Table S4. Assorted band assignments of urethane.

Wavenumber (cm^{-1})	Band assignment	Intensity	Molecular structure	References
3453	ν NH	w	non H-bonded	[1]
3381	ν NH, Ar-O-C(=O)-NH-R	m	H-bonded	[2,3]
3330-3320	ν NH	w	H-bonded	[1]
3280	ν NH	sh, w	H-bonded	[1]
3060	ν CH	vw	aromatic ring	
1715-1690	ν C=O	s	aliphatic-aromatic H-bond, non H-bond	[1,4,5,8–11]
1530	γ N-H+ν C-N, Ar-O-C(=O)-NH-R	s-m	secondary urethanes	[1,4,6,13]
1506	ν C-C, ring	m	secondary urethanes, associated or in solid phase	[4]
1470	γ NH + ν CN, γ CH aliphatic, Ar-O-C(=O)-NH-R	m	γ CH_2 + ω CH_2	[7]
1387	δ_s CH, $\delta_{s,\ i.p.}$ CH	vw	CH_3, compare to Polycarbonate	
1220	ν CN, ν CO	s-m	secondary urethanes, associated or in solid phase	[7,12,13]
1170	ν as COC	s		[7,13]
1080	ν COC	m	ν s C-O-C aliphatic,	[7,13]
1020	ν COC, ν CN, aliphatic, Ar-O-C(=O)-NH-R	m		[7,13]

1. McCarthy, S.J.; Meijs, G.F.; Mitchell, N.; Guntillake, P.A.; Heath, G.; Brandwood, A.; Schindhelm, K. *Biomaterials* **1997**, *18*, 1387–1409.
2. Yu, S.; Ma, M.; Liu, J.; Tao, J.; Liu M.; Gao, C. *J. Membr. Sci.* **2011**, *379*, 164–173.
3. Li, M.-S.; Ma, C.-C.M.; Lin, M.-L.; Lu, M.-S.; Chen, J.-L.; Chang, F.-C. *Polymer* **1997**, *38*, 845–853.
4. Lin, M.-L.; Chang, K.-H.; Chang, F.-C.; Li, M.-S.; Ma, C.-C.A. *J. Polym. Sci. Part B Polym. Phys.* **1997**, *35*, 2183–2191.
5. Günzler, H.; Gremlich, H.-U. *IR-Spektroskopie - Eine Einführung*; Wiley-VCH: Weinheim, Germany, 2000.
6. Lee, S.-N.; Stolarski, V.; Letton, A.; Laane, J. *J. Mol. Struc.* **2000**, *521*, 19–23.
7. Mendoza-Payan, J.G.; Gallardo, S.F.; Marquez-Lucero, A. *Sens. Actuators B* **2009**, *142*, 130–140.
8. Coleman, M.M.; Lee, K.H.; Skrovanek, D.J.; Painter, P.C. *Macromolecules* **1986**, *19*, 2149–2157.
9. Corcuera, M.A.; Rueda, L.; Fernandez d'Arlas, B.; Arbelaiz, A.; Marieta, C.; Mondragon, I.; Ecaiza, A. *Polym. Degrad. Stab.* **2010**, *95*, 2175–2184.
10. Coleman, M.M.; Skrovanek, D.J.; Hu, J.; Painter, P.C. *Macromolecules* **1988**, *21*, 59–65.
11. Dechant, J. *Ultrarotspektroskopische Untersuchungen an Polymeren*; Akademie-Verlag: Berlin, Germany, 1972.
12. Nyquist, R.A.; Potts, W.J. *Spectrochim. Acta* **1961**, *17*, 679–697.
13. Socrates, G. *Infrared and Raman Chracteristic Group Frequencies*, 3rd Ed.; Wiley: Hoboken, NJ, USA, 2001.
